# The use of prescription drugs and health care services during the 6-month post-COVID-19 period

**DOI:** 10.1038/s41598-023-38691-9

**Published:** 2023-07-19

**Authors:** Kerli Mooses, Kaarel Vesilind, Marek Oja, Sirli Tamm, Markus Haug, Ruth Kalda, Kadri Suija, Anna Tisler, Tatjana Meister, Maria Malk, Anneli Uusküla, Raivo Kolde

**Affiliations:** 1 Institute of Computer Science, University of Tartu, Tartu, Estonia; 2 Institute of Family Medicine and Public Health, University of Tartu, Tartu, Estonia

**Keywords:** Viral infection, Influenza virus, Epidemiology, Signs and symptoms, Scientific data

## Abstract

COVID-19 and other acute respiratory viruses can have a long-term impact on health. We aimed to assess the common features and differences in the post-acute phase of COVID-19 compared with other non-chronic respiratory infections (RESP) using population-based electronic health data. We applied the self-controlled case series method where prescription drugs and health care utilisation were used as indicators of health outcomes during the six-month-long post-acute period. The incidence rate ratios of COVID-19 and RESP groups were compared. The analysis included 146 314 individuals. Out of 5452 drugs analysed, 14 had increased administration after COVID-19 with drugs for cardiovascular diseases (trimetazidine, metoprolol, rosuvastatin) and psychotropic drugs (alprazolam, zolpidem, melatonin) being most prevalent. The health impact of COVID-19 was more apparent among females and individuals with non-severe COVID-19. The increased risk of exacerbating pre-existing conditions was observed for the COVID-19 group. COVID-19 vaccination did not have effect on drug prescriptions but lowered the health care utilisation during post-acute period. Compared with RESP, COVID-19 increased the use of outpatient services during the post-infection period. The long-term negative impact of COVID-19 on life quality must be acknowledged, and supportive health care and public health services provided.

## Introduction

The COVID-19 pandemic caused by the SARS-CoV-2 virus is a great challenge for the whole society, especially for the healthcare system, as the severity and the spread of the virus have increased the need for healthcare services. At the same time, the lockdowns and other restrictions implemented to reduce social contact have had a negative impact on economy^1^ as well as on mental^2,3^ and physical health of individuals^3–5^. It is now well established that COVID-19 is not limited to the acute phase. There is an extensive body of evidence that COVID-19 can have a long-lasting impact on patients’ health^5–8^ and can be attributed to increased mortality during the post-COVID period^9^. Having persistent symptoms or a new onset of symptoms after the acute phase of COVID-19 is called post-COVID-19 condition^10,11^ or long-COVID^12,13^. According to a review, patients can experience post-COVID-19 condition regardless of the severity of the initial illness^12^. There is some evidence suggesting that the length of post-acute sequelae is longer and the risk for re-hospitalisation is higher for patients who were hospitalised with COVID-19 compared to patients who did not need inpatient care^14–18^. However, the lack of research involving patients who had mild COVID-19 and did not require hospitalisation has previously been pointed out^7,12^. Moreover, for a long time, there was no consensus on the definition of post-COVID-19 condition^12,19^. As a result, discrepancies in the follow-up period ranging from 4 weeks to 3 months after the onset of COVID-19 can be observed^6,13^. More recently, WHO described that post-COVID-19 condition usually arises 3 months from the onset of COVID-19, and symptoms last at least 2 months^20^. Thus, there is a need for studies focusing on symptoms extending beyond 3 months to better understand the long-term effects of COVID-19^7^.

The occurrence of chronic sequelae is not unique only for the SARS-CoV-2 virus as post-acute infection syndrome (PAIS) has been associated with different viral, bacterial, and parasitic infections^21^. To date, it has been shown that although some symptoms of PAIS are trigger-specific, there is a significant overlap in the symptoms, with the most prevalent being fatigue, flu-like symptoms (e.g., fever, muscle pain, sweating), neurological (e.g., impaired concentration), and rheumatologic symptoms (e.g., joint and muscle pain)^21^. Similar symptoms have also been reported for post-COVID-19 condition^10,12,13,22–25^. It is most likely that SARS and SARS-CoV-2 viruses are not the only viruses that can cause long-term chronic health problems, and PAIS could be present for other acute respiratory diseases such as influenza^21,26,27^ or respiratory syncytial virus infections^27^. However, to date, PAIS is understudied, and there is a need for cohorts with well-documented infectious triggers^21^.

Much of the research on SARS-CoV-2 sequelae has been performed on large claims and electronic health record databases^17,28–34^. However, the information on specific symptoms is often not reliably recorded in administrative datasets^29,32^, and different surrogate markers such as prescriptions and health care contacts can be considered valid alternatives to evaluate the occurrence of disease or symptoms^17,35,36^. We take an untargeted approach, considering all possible prescription drugs, to discover novel associations with COVID-19 while comparing with other acute respiratory diseases, to highlight the unique aspects of COVID-19, and reduce technical artefacts caused by the underlying distribution of data.

## Methods

In this population-based study, we combined individual-level data from national electronic health databases to assess the effect of COVID-19 and other non-chronic respiratory infectious diseases during the post-infection period. The study period was 01.02.2018–30.03.2022. The Research Ethics Committee of the University of Tartu approved our study (No. 330/T-10) and waived the requirement for informed consent. All methods were performed in accordance with the relevant guidelines and regulations.

### Data

This study is nested in the population-based e-health records (EHR), health claims, and prescriptions data from the universal tax-funded healthcare system in Estonia. EHR stores data for case summaries, vaccinations, lab tests, etc., for all people in Estonia and receives this data from all general practitioners and hospitals, both private and state-owned. Health claims include information for 95% of population^37^ about health care utilisations (provided service, date of service, treatment type, ICD-10 diagnoses), while the prescriptions database stores detailed information about all issued and purchased drugs and vaccines^38^. Individual-level linkage of these databases was performed using a unique personal code given to all persons living in Estonia. Data was mapped to the Observational Medical Outcomes Partnership (OHDIS OMOP) common data model version 5.3^39^. The mapping process and detailed description of the databases have been previously described by Oja et al.^40^.

### Design

**Figure 1:**
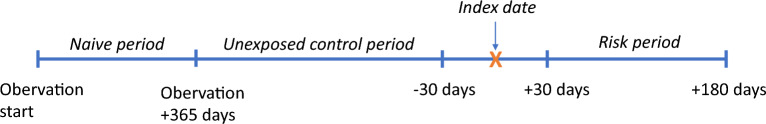
Control and risk periods in the study.

We applied the self-controlled case series (SCCS) method^41^, where individuals act as their own controls, reducing the influence of confounding variables. The index date was the date of the diagnosis of interest (COVID-19 or non-chronic respiratory infectious disease). The risk period was 30–180 days after the index date to represent the post-acute period of the disease (Fig. 1). We compared the outcome incidence rates from this period with a control period from the start of the observation period to 30 days before the index date. The observation period start is 01.02.2018.

To account for possible technical biases, we introduced a comparator group of events where we performed identical analyses. For example, with prescriptions, an “anchoring bias”^42^ can occur, where drugs not related to COVID-19 are also prescribed during the visit where the diagnosis is given, affecting the prescription rate on the risk period after the visit. However, such bias applies to both COVID-19 and the comparator group, leading to similar changes in incidence rates. To identify COVID-19-specific effects, we concentrate on results where the incidence rate ratio difference between COVID-19 and the comparator is statistically significant.

### Cases

The COVID-19 group consisted of adult individuals with at least one positive SARS-CoV-2 test result confirmed by real-time polymerase chain reaction (PCR) or SARS-CoV-2 antigen testing on nasopharyngeal specimens. The index date was the first positive SARS-CoV-2 test result between 01.02.2020 and 30.09.2021.

The comparator group comprised adults diagnosed with the non-chronic respiratory infectious disease, but no positive SARS-CoV-2 test during the observation period (RESP group). The selection process of respiratory infectious diseases that were included in the study was following: first, all respiratory findings were selected and sorted based on the occurrence, then codes with no occurrence in our database and all chronic conditions (e.g., asthma and allergy-related findings, chronic obstructive lung disease, chronic sinusitis, etc.) were excluded. The list of diagnoses included in the study is presented in Supplementary table 1. Influenza accounted for 1.5% of all cases included. The index date of RESP exposure was the first occurrence of non-chronic respiratory infectious disease between 01.02.2019 and 30.09.2021.

For both COVID-19 and RESP cases, continuous observation for 1 year before and 6 months after the index date was required (Fig. 1). The acute phase of the disease was 30 days after the positive SARS-CoV-2 test for COVID-19 cases and diagnosis of respiratory infectious disease for RESP cases. For all cases, the unexposed control period was 180–30 days prior to the index date, and the risk period was 30 days–6 months after the index date. The risk period can be considered as the post-acute infection period.

### Measures

For both COVID-19 and RESP groups, several subgroups were created based on sex (male/female), age (18–39 years/40–64 years/65 and older), hospitalisation status during the acute phase of the disease (yes/no), and Charlson Comorbidity Index (CCI) (CCI = 0/ CCI > 0). For only COVID-19 cases, vaccination status (yes/no) during the index date was created. Sex stratification was created as sex differences in post-COVID-19 symptoms have been reported previously^43^. The subject was classified as hospitalised due to the index event if the individual had an inpatient or emergency room visit between 2 weeks before and 30 days after the index date^44^. CCI was used to assess the presence of comorbid conditions and computed based on the health data before the index date^45,46^. Based on the CCI, subjects were divided into two groups—healthy subjects (CCI = 0) and subjects with at least one comorbid condition (CCI > 0). For COVID-19 cases, the subject was considered vaccinated against COVID-19 when at least one COVID-19 vaccine was received 6 months to 14 days before the positive SARS-CoV-2 test result (index date).

### Main outcomes

The main interest were incident and recurrent health outcomes and health care utilisation 30 to 180 days after COVID-19 or RESP. We used prescription data as a surrogate marker for health outcomes which can be considered a valid alternative to evaluate the occurrence of disease or symptoms^17,35,36^. A total of 5 452 different drugs at the ingredient level were included in the analysis using the 5th level of Anatomical Therapeutic Chemical (ATC) codes^24^. We defined incident episodes of specific drug when the drug had not been prescribed within 12 months prior to the index date. When the specific drug was prescribed within 12 months prior to the index date and also after the exposure event, it was classified as recurrent use. We created groups for each drug in the ATC code list using CAPR package^47^ in R (v 4.0). Drug groups with less than 20 subjects were excluded from subsequent analysis. In the “Results” section, we focus on drugs where a statistically significant increase in use was present in the COVID-19 group compared to the RESP group (p > 0.05).

Regarding health care utilisation, hospitalisation, and outpatient visits were analysed separately. All hospitalisations and outpatient visits, irrespective of the cause, were included.

### *Dataanalysis*

For descriptive analysis, we used proportions or means and standard deviations where appropriate. The standardised mean difference was used to compare the distribution of baseline covariates between Covid-19 and RESP groups. The standardised mean difference less than 0.2 indicates that the difference between groups is small^48^. In applying the SCCS method, we estimated incidence rate ratios and 95% confidence intervals for each outcome of interest using Poisson regression and controlled for age using a spline function, as implemented in the SelfControlledCaseSeries package^49^ by OHDSI in R (v 4.0). We performed identical analyses on both groups—COVID-19 and RESP—and compared the resulting incidence rate ratio estimates using Z-test on the difference between the estimates. As we tested all ATC codes simultaneously, we corrected the resulting p-values using Bonferroni correction.

## Results

Our study included 146 314 individuals, of which 56.8% were female (Supplementary table 3). COVID-19 and RESP groups were similar in terms of age, sex, and health status in the pre-acute period (Table 1).

**Table 1:** Description of COVID-19 and non-chronic respiratory infectious disease (RESP) group.

	COVID-19 group (n = 77 466)	RESP group (n = 68 848)	Standardised mean difference
Socio-demographic characteristics			
Age (years)	46.7 ± 17.4	47.9 ± 18.1	0.05
Sex: Female	42,512 (54.9%)	40,612 (59.0%)	0.08
Health status in pre-acute period			
Charlson Comorbidity Index > 0	25,915 (33.5%)	23,417 (34.0%)	0.01
Charlson Comorbidity Index	0.7 ± 1.8	0.7 ± 1.7	0.00
Medical history^a^			
Heart disease	14,570 (18.8%)	15,489 (22.5%)	0.09
Heart failure	4976 (6.4%)	5341 (7.8%)	0.05
Ischemic heart disease	2326 (3.0%)	2823 (4.1%)	0.06
Malignant neoplastic disease	2726 (3.5%)	2956 (4.3%)	0.04
Diabetes mellitus	4238 (5.5%)	4007 (5.8%)	0.02
Depressive disorder	4238 (5.5%)	5751 (8.4%)	0.11
Hypertensive disorder	12,620 (16.3%)	12,174 (17.7%)	0.04
Human immunodeficiency virus infection	353 (0.5%)	398 (0.6%)	0.02
Obesity	3327 (4.3%)	2890 (4.2%)	0.00
Acute period of infection			
Hospitalisation during index event	5239 (6.8%)	2670 (3.9%)	0.13
Post-acute period of infection			
Hospitalisation during risk period	3627 (4.7%)	3682 (5.3%)	0.03
Outpatient visits during risk period	64,850 (83.7%)	57,093 (82.9%)	0.02

^a^The ICD-10 codes for medical history conditions are in Supplementary table 2.

**Figure 2:**
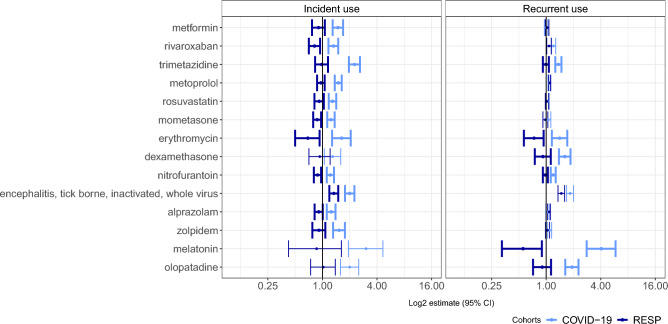
Adjusted incident rate ratios comparing incident and recurrent use of drugs in COVID-19 and RESP group in Estonia. Statistically significant differences according to Bonferroni correction between groups are presented in bold error bars.

Out of 5452 drug ingredients included in the analysis, 14 drugs had increased administration after COVID-19 compared to the RESP group (Fig. 2, Supplementary table 4). Most of the drugs that use had increased in the post-COVID-10 period were administered for the first time (78.6%) and were indicated for the use of cardiovascular diseases (trimetazidine, metoprolol, rosuvastatin) or were psychotropic drugs (21.4%) (alprazolam, zolpidem, melatonin).

**Figure 3:**
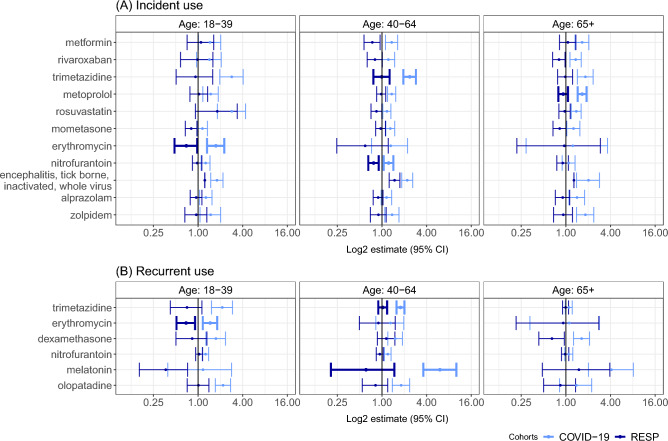
Adjusted incidence rate ratios comparing incident (**A**) and recurrent (**B**) drug use in COVID-19 and RESP subgroups of Charlson Comorbidity Index (Charlson = 0 and Charlson > 0), hospitalisation during acute infection phase (hospitalised and non-hospitalised) and sex (male and female) in Estonia. Statistically significant differences between groups are presented in bold error bars.

As for the subgroups, there was no significant increase in incident drug administration for subjects who were hospitalised with COVID-19 and for males compared with RESP cases (Fig. 3, Supplementary tables 5–7). The impact of COVID-19 on health was more apparent among females and individuals with non-severe COVID-19. Individuals with concomitant chronic diseases (Charlson > 0) experienced more health events requiring recurrent medications than those without the mortality-predicting disease.

**Figure 4:**
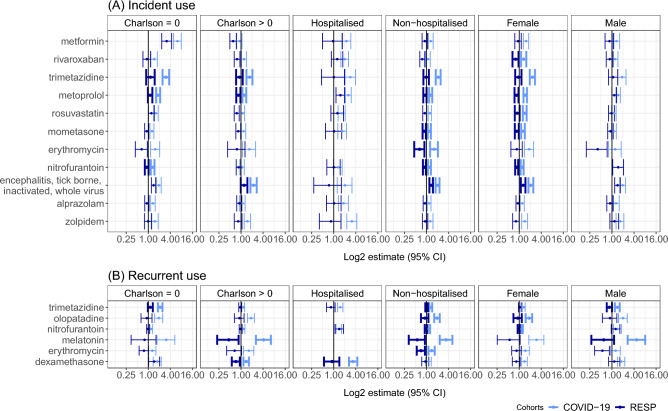
Adjusted incidence rate ratios comparing incident (**A**) and recurrent (**B**) drug use in age groups in COVID-19 and RESP groups in Estonia. Statistically significant differences between groups are presented in bold error bars.

Stratified analysis by age group showed that COVID-19 had the strongest impact on drug prescriptions in the age group 40–64 (Fig. 4, Supplementary table 8). In the oldest age group (65 and older), only metoprolol had a significant increase in incident use compared to the RESP group (1.66, 95% CI 1.44–1.90 vs 0.92, 95% CI 0.79–1.07). In the age group 18–39 years, the COVID-19 group had a higher incident and recurrent use of erythromycin than the RESP cohort.

Before the onset of COVID-19, 3.5% were vaccinated against SARS-CoV-2 with at least one vaccination dose. Vaccination status during the onset of COVID-19 did not affect post-COVID-19 period drug prescriptions. Vaccinated individuals had a significantly lower number of inpatient and outpatient visits during the post-COVID-19 period compared with those who were not vaccinated (inpatient: IRR 0.84, 95% CI 0.70–0.99 vs 1.78, 95% CI 1.14–1.22; outpatient: IRR 0.92, 95% CI 0.90–0.95 vs 1.16, 95% CI 1.15–1.16).

**Figure 5:**
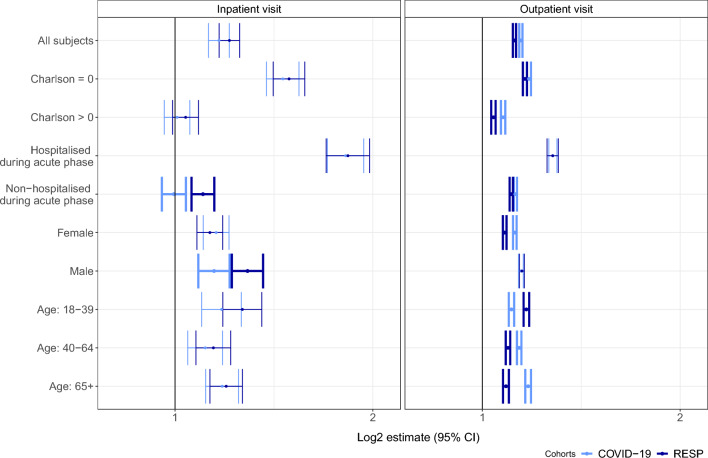
Adjusted incidence rate ratios comparing inpatient and outpatient visits of COVID-19 and RESP group in main and subgroups during the post-COVID-19 period in Estonia. Statistically significant differences between groups are presented in bold error bars.

Having had RESP or COVID-19 led to an increased number of hospitalisation and outpatient health care episodes compared to the pre-acute infection period. Compared to the RESP group, those with COVID-19 were more likely to use out-patient services during the post-infection period, with a stronger effect among women, those with non-severe COVID-19, and older than 40 years (Fig. 5, Supplementary table 9). COVID-19 was significantly associated with lower intensity of outpatient visits for 18–39 year-olds (1.11, 95% CI 1.10–1.12 vs 1.17, 95% CI 1.16–1.18) and inpatient visits for males (1.15, 95% CI 1.08–1.21 vs 1.29, 95%CI 1.22–1.36) and individuals who were not hospitalised during the acute phase of the virus (1.00, 95% CI 0.95–1.04 vs 1.83, 95% CI 1.7–1.98) compared with RESP.

## Discussion

This study set out to investigate the effect of COVID-19 infection on drug administration and healthcare utilisation in the post-COVID-19 period compared with other respiratory diseases and the period before the infection. This comparative approach allowed us to identify common features and differences between COVID-19 and other acute respiratory infections in the same setting (epidemiological, health care, time) and provide indications of characteristics distinctive to COVID-19. We showed that compared with other respiratory diseases, COVID-19 exhibited increased risks of new onset of health problems (especially cardiovascular conditions and the use of psychotropic and immunomodulating drugs), exacerbation of pre-existing conditions, and increased outpatient health care use.

Our results showed that both COVID-19 and other respiratory diseases increased inpatient and outpatient visits during the post-acute period, whereas the readmission rates were remarkably higher for individuals hospitalised with acute infection compared to those with non-severe infection. Higher readmission rates for COVID-19 patients have also been reported earlier^32,50^. However, contrary to previous studies^17,32,51^, we did not find excess incident drug use or healthcare utilisation in individuals hospitalised with COVID-19 compared with other respiratory diseases. Therefore, our findings suggest that in case of severe respiratory disease, COVID-19 and other respiratory diseases have similar long-term health effect. As for mild COVID-19, our results indicate excess long-term health consequences, which are consistent with existing literature^32,33,35^. We observed the increased incident and recurrent drug use for several drugs and excess outpatient visits in COVID-19 patients compared with other respiratory diseases and the pre-acute infection period. These findings highlight that COVID-19 puts an extra burden on the healthcare system as addition to the workload during the outbreaks of COVID-19, mild COVID-19 cases can result in long-term health problems that must be dealt with. This is important aspect that should be kept in mind when creating health policies and allocating resources for the health sector.

Several covariates like age, gender and existing health conditions can affect health outcomes, which is also the case for COVID-19. A growing body of literature indicates a higher prevalence and risk of post-COVID-19 among females compared to males^25,43,52^. Moreover, older age and concomitant chronic diseases are previously associated with the risk of post-COVID-19^6,51^. Concerning age, our results are in accordance with previous studies^6,51^ as we observed increased incident use for several drugs and post-COVID-19 outpatient visits for females, whereas no increase was present for males. As for age, only the prescribing of a few active ingredients had increased during the post-COVID-19 period, indicating that the effect of age could be modest. In contrast to earlier findings^6,51^, we did not observe differences in drug prescription rates during the post-COVID-19 period between the individuals with and without existing comorbidities. Our findings suggest that COVID-19 has a negative long-term impact on health despite the overall health status of an individual.

Our study found a significant increase in incident cardiovascular drug use during the post-COVID-19 period compared with other respiratory diseases. This is in line with previous studies focusing on drug use^17^ or cardiovascular incidence^33,51,53^ after COVID-19 infection. In accordance with the present results, previous studies have also demonstrated that COVID-19 increases the risk of cardiovascular incidence regardless of existing comorbidities^25,33^ or the severity of the acute phase of COVID-19^16,31,33^. Another set of drugs in our study that had increased incident rate were several psychotropic drugs, mainly sedatives. Different psychotic disorders, like anxiety, mood and sleep disorders, are common symptoms of post-COVID-19^12,30,31,51^. Despite this, the previous findings on incident psychotropic drug use are contradictory. For example, a Danish population-based study did not find an increase in incident use of psychotropic drugs^35^ while a study in the USA, including mainly male participants, reported an excess incident use of antidepressants and sedatives^17^. In contrast to earlier findings, we had a significant increase in incident use of several psychotropic drugs for the whole COVID-19 group compared with other respiratory diseases and for females but not for males. The overall increase in incident sedative use could be linked to the fear and anxiety caused by the pandemics and the increase in economic instability^54^. It could be argued that the increased use of sedatives among females can be partially attributed to gender inequalities which have increased during the COVID-19 lockdown and put more responsibilities, like parenting and domestic shores, on employed women^55,56^. At the same time, some of the differences between current and previous studies could be attributed to the prescription practice in different countries, and thus, more detailed analysis is needed.

We observed increased incident use of topical mometasone, indicating persistent olfactory dysfunction during the post-COVID-19 period. Loss of smell is one typical symptom of COVID-19 and a common problem during the post-acute phase^57^. Although some evidence is emerging that topical mometasone has no effect on olfactory dysfunction recovery^58,59^, it has been a recommended practice that has also been applied to our sample. Also, compared with other respiratory diseases, higher use of dexamethasone was present for COVID-19 patients who had previously used this drug and had concomitant chronic diseases or were hospitalised during the acute phase of infection. Corticosteroids, including dexamethasone, is often used in case of post-COVID-19 pneumonia or other post-COVID-19 lung disease^60,61^. Our results indicate the detrimental effect of COVID-19 on lungs. For several drugs that had increased use in our study, such as metformin^62,63^, rivaroxaban^64,65^, trimetazidine^66,67^, metoprolol^68^, rosuvastatin^69^, a beneficial effect during the acute phase of COVID-19 has been suggested. As for their effect during post COVID-19 period less is known. For example, there is some evidence that metformin^63^ and metoprolol^70^ could contribute to the treatment of post-COVID-19 patients while the need for additional studies has been stressed^63^. One novel finding of our study was the increased use of erythromycin, an antibiotic used to treat different skin conditions. The increased use of this antibiotic could be related to the previous findings that face masks worn during the COVID-19 restrictions cause several skin diseases, including acne^71,72^. Surprisingly, we observed increased vaccination against encephalitis after COVID-19. It could be hypothesised that it is partially associated with the increase in outpatient visits—as one is already visiting a doctor due to some COVID-19-related health problem, it is convenient to update one’s vaccinations. In addition, it could be argued that increased health anxiety or the higher use of outdoor spaces and nature tracks during COVID-19 restrictions^73,74^ could have some impact on the vaccination behaviour against encephalitis. However, more research is needed on this topic.

One thing that could protect against the negative impact of post-COVID-19 condition is the vaccination^28,29,75^. Today the studies focusing on the vaccination effect on post-COVID-19 condition are scarce^75^, which makes it impossible to draw solid conclusions. Some studies have reported a reduced risk of sequelae for some post-COVID-19 symptoms but not for all^28,29^. In the current study, we did not detect any protective effect of vaccination on drug use, however, a reduction in healthcare utilisation among vaccinated individuals was present during the post-COVID-19 period. It should be kept in mind that the proportion of vaccinated individuals in the current study was relatively low, as the vaccination started at the end of 2020 and at first among selected groups, such as healthcare workers, older people, and those in risk groups. Therefore, more research is needed on the long-term effect of vaccination.

The current study also has some limitations that must be acknowledged. Although our approach identifies the incident post-acute sequelae in patients with COVID-19 and other respiratory diseases, it does not delineate which sequelae may be direct or indirect consequences of COVID-19 infection. We tried to mitigate this by using the pre-post design. Our database has excellent coverage of prescription drugs, but we do not have any information on the use of over-the-counter drugs like paracetamol, ibuprofen and others. This could leave unnoticed some milder cases of post-COVID-19 syndrome. Moreover, in our analysis, we did not take into account the dosage, which means that the increase in recurrent drugs due to the increase in dosage is not detected. Therefore, the changes in recurrent drug use could be somewhat underestimated. It should be kept in mind that using prescribed medications to approximate health conditions warrant careful interpretation. As for the health care utilisation, there is a possibility that the 6-month follow-up period is too short of capturing all individuals who have referrals to health specialists as due to the increased workload of health care service providers, some appointments could be postponed beyond the current follow-up period. Thus, an extended follow-up period in further studies should be considered. In addition, the COVID-19 cohort consists of subjects who had a confirmed PCR test and those who had positive COVID-19 using only the over-the-counter testing were not included into the cohort. However, we believe that this has very minor effect on the results as during the study period it was required in Estonia that all over-the-counter tests are confirmed with PCR test in order to receive the COVID certificate. Having this certificate reduced the national restrictions and allowed traveling in European Union. At the same time, the good coverage of our healthcare databases and large-scale testing provided for both symptomatic and asymptomatic people during the pandemic, including individuals hospitalised due to COVID-19 and individuals with mild or asymptomatic COVID-19 in the analysis, are some strengths of the current study. Moreover, the inclusion of control group with other respiratory diseases helps better to understand the unique long-term effect of COVID-19 on health and adds invaluable information to the existing knowledge on post-COVID-19 condition.

Overall, our findings indicate increased health problems after COVID-19 which put an additional burden on healthcare system. Based on post-COVID-19 drug administration and healthcare utilisation we claim that health problems that need extra medical attention are present, however, they rather tend to be mild and not require hospital admission. The long-term negative impact of COVID-19 on life quality must be acknowledged, and supportive health care and public health services provided.

## Supplementary Information

Supplementary Tables.

## Data Availability

There are legal restrictions on sharing a de-identified data. According to legislative regulation and data protection law in Estonia, the authors cannot publicly release the data received from the health data registers in Estonia. The data can be requested by completing the application in order to carry out research or an evaluation of public interest (https://www.tehik.ee/en/statistics). More information about data availability: Kerli.Mooses@ut.ee.
