# Supplementary material for: The use of prescription drugs and health care services during the 6-month post-COVID-19 period
Source: Sci Rep. 2023 Jul 19;13:11638. doi: 10.1038/s41598-023-38691-9 (PMC10356787; doi:10.1038/s41598-023-38691-9)
Supplement: Supplementary file 1 — Supplementary Tables. [file 41598_2023_38691_MOESM1_ESM.docx]

**The use of prescription drugs and health care services during the six-month post-COVID-19 period**

Kerli Mooses*^1^, Kaarel Vesilind^1^, Marek Oja^1^, Sirli Tamm^1^, Markus Haug^1^, Ruth Kalda^2^, Kadri Suija^2^, Anna Tisler^2^, Tatjana Meister^2^, Maria Malk^1^, Anneli Uusküla^2^, Raivo Kolde^1^

^1^ Institute of Computer Science, University of Tartu

^2^ Institute of Family Medicine and Public Health, University of Tartu

**Supplementary table 1.** SNOMED CT codes for non-chronic respiratory infectious disease cohort

| **SNOMED code** | **Name** |
| --- | --- |
| 10685111000119100 | Upper respiratory tract infection due to Influenza |
| 719590007 | Influenza caused by seasonal influenza virus |
| 70076002 | Rhinitis |
| 85469005 | Hypostatic pneumonia |
| 195742007 | Acute lower respiratory tract infection |
| 6142004 | Influenza |
| 59707005 | Abscess of pharynx |
| 41582007 | Streptococcal tonsillitis |
| 415125002 | Pneumocystosis pneumonia |
| 276443001 | Acute laryngitis and/or tracheitis |
| 278516003 | Lobar pneumonia |
| 195728004 | Acute bronchitis due to rhinovirus |
| 195726000 | Acute parainfluenza virus bronchitis |
| 195720006 | Acute streptococcal bronchitis |
| 195727009 | Acute respiratory syncytial virus bronchitis |
| 195721005 | Acute haemophilus influenzae bronchitis |
| 29608009 | Acute epiglottitis |
| 186675001 | Viral pharyngoconjunctivitis |
| 233599001 | Acute mycoplasmal bronchitis |
| 233613009 | Fungal pneumonia |
| 196112005 | Abscess of lung with pneumonia |
| 312342009 | Infective pneumonia |
| 46970008 | Mycoplasma pneumonia |
| 64917006 | Parainfluenza virus pneumonia |
| 196019004 | Bronchitis and pneumonitis due to chemical fumes |
| 26650005 | Acute tracheitis |
| 195881003 | Pneumonia due to respiratory syncytial virus |
| 61700007 | Influenza with non-respiratory manifestation |
| 398447004 | Severe acute respiratory syndrome |
| 75570004 | Viral pneumonia |
| 34020007 | Pneumonia due to Streptococcus |
| 70036007 | Haemophilus influenzae pneumonia |
| 51530003 | Pneumonia due to Escherichia coli |
| 82272006 | Common cold |
| 10509002 | Acute bronchitis |
| 71186008 | Croup |
| 5505005 | Acute bronchiolitis |
| 22754005 | Staphylococcal pneumonia |
| 233607000 | Pneumococcal pneumonia |
| 430395005 | Pneumonia due to Gram negative bacteria |
| 53084003 | Bacterial pneumonia |
| 54398005 | Acute upper respiratory infection |
| 73452002 | Abscess of lung |
| 195878008 | Pneumonia and influenza |
| 396285007 | Bronchopneumonia |
| 32398004 | Bronchitis |
| 233604007 | Pneumonia |
| 49727002 | Cough |
| 41207000 | Adenoviral pneumonia |
| 233609002 | Pneumonia caused by Chlamydiaceae |
| 195739001 | Acute bronchiolitis due to respiratory syncytial virus |
| 64479007 | Pneumonia due to Klebsiella pneumoniae |
| 205237003 | Pneumonitis |
| 7678002 | Cytomegaloviral pneumonia |
| 195886008 | Group B streptococcal pneumonia |
| 41381004 | Pneumonia due to Pseudomonas |
| 64375000 | Acute laryngotracheitis |
| 6655004 | Acute laryngitis |
| 43878008 | Streptococcal sore throat |
| 363746003 | Acute pharyngitis |
| 17741008 | Acute tonsillitis |
| 55355000 | Acute laryngopharyngitis |
| 232403001 | Chlamydial pharyngitis |

**Supplementary table 2.** ICD-10 codes for medical history diagnoses presented in Table 1.

| **Medical history diagnoses** | **ICD-10 code** |
| --- | --- |
| Heart disease | I01, I05-I09, I11, I13, I20-I22, I24, I25, I27, I33-I36, I38-I52, Q20-Q24, S26 |
| Heart failure | I50 |
| Ischemic heart disease | I20, I21, I22, I24, I25 |
| Malignant neoplastic disease | B21, C00-C26, C30-C34, C37-C41, C43-C58, C60-C85, C88, C90-C97, D45, D46 |
| Diabetes mellitus | E10-E14, O24 |
| Depressive disorder | F32, F33 |
| Hypertensive disorder | I10, I15, O10, O11, O13, O14, O16 |
| Human immunodeficiency virus infection | B20-B24, Z21 |
| Obesity | E65, E66 |

**Supplementary table 3.** Number of exposed to drug and healthcare use by subgroups

|  | **ATC code** | **All** | | **Charlson** | | | | **Hospitalised during acute phase** | | | | **Sex** | | | | **Age** | | | | | |
| --- | --- | --- | --- | --- | --- | --- | --- | --- | --- | --- | --- | --- | --- | --- | --- | --- | --- | --- | --- | --- | --- |
|  |  |  |  | **0** | | **>1** | | **Yes** | | **No** | | **Male** | | **Female** | | **18-39** | | **40-64** | | **65+** | |
| **COVID-19** |  |  | |  | |  | |  | |  | |  | |  | |  | |  | |  | |
| Metformin | A10BA02 | 5040 | 567 | | 4473 | | 1003 | | 4037 | | 1929 | | 3111 | | 386 | | 2274 | | 2380 | |  |
| Rivaroxaban | B01AF01 | 3778 | 1241 | | 2537 | | 1081 | | 2697 | | 1714 | | 2064 | | 279 | | 1408 | | 2091 | |  |
| Trimetazidine | C01EB15 | 2646 | 876 | | 1770 | | 433 | | 2213 | | 720 | | 1926 | | 258 | | 1190 | | 1198 | |  |
| Metoprolol | C07AB02 | 10 877 | 3561 | | 7316 | | 2065 | | 8812 | | 3951 | | 6926 | | 825 | | 4679 | | 5373 | |  |
| Rosuvastatin | C10AA07 | 6886 | 2264 | | 4622 | | 1087 | | 5799 | | 2780 | | 4106 | | 163 | | 3604 | | 3119 | |  |
| Mometasone | D07AC13 | 7989 | 4397 | | 3592 | | 658 | | 7331 | | 2860 | | 5129 | | 2746 | | 3623 | | 1620 | |  |
| Erythromycin | D10AF02 | 955 | 671 | | 284 | | 35 | | 920 | | 373 | | 582 | | 731 | | 193 | | 31 | |  |
| Dexamethasone | H02AB02 | 1568 | 663 | | 905 | | 436 | | 1132 | | 617 | | 951 | | 270 | | 814 | | 484 | |  |
| Nitrofurantoin | J01XE01 | 8211 | 4644 | | 3567 | | 735 | | 7476 | | 461 | | 7750 | | 3255 | | 3159 | | 1797 | |  |
| Encephalitis, tick borne, inactivated, whole virus | J07BA01 | 3628 | 2432 | | 1196 | | 209 | | 3419 | | 1495 | | 2133 | | 1529 | | 1698 | | 401 | |  |
| Alprazolam | N05BA12 | 6508 | 3338 | | 3170 | | 669 | | 5839 | | 1663 | | 4845 | | 1642 | | 3309 | | 1557 | |  |
| Zolpidem | N05CF02 | 2738 | 1186 | | 1552 | | 386 | | 2352 | | 866 | | 1872 | | 499 | | 1398 | | 841 | |  |
| Melatonin | N05CH01 | 213 | 90 | | 123 | | 30 | | 183 | | 80 | | 133 | | 65 | | 93 | | 55 | |  |
| Olopatadine | S01GX09 | 708 | 317 | | 391 | | 39 | | 669 | | 232 | | 476 | | 340 | | 272 | | 96 | |  |
| Inpatient visits |  | 26 238 | 12 388 | | 13 850 | | 5239 | | 20 999 | | 9532 | | 16 706 | | 8806 | | 10 102 | | 7330 | |  |
| Outpatient visits |  | 77 466 | 51 551 | | 25 915 | | 5239 | | 72 227 | | 34 954 | | 42 512 | | 30 033 | | 34 675 | | 12 758 | |  |
| **RESP** |  |  |  | |  | |  | |  | |  | |  | |  | |  | |  | |  |
| Metformin | A10BA02 | 4652 | 680 | | 3972 | | 384 | | 4268 | | 1725 | | 2927 | | 768 | | 1999 | | 2269 | |  |
| Rivaroxaban | B01AF01 | 3520 | 1160 | | 2360 | | 480 | | 3040 | | 1502 | | 2018 | | 474 | | 1209 | | 2074 | |  |
| Trimetazidine | C01EB15 | 2934 | 947 | | 1987 | | 190 | | 2744 | | 782 | | 2152 | | 372 | | 1109 | | 1639 | |  |
| Metoprolol | C07AB02 | 12 072 | 4247 | | 7825 | | 1183 | | 10889 | | 4211 | | 7861 | | 1706 | | 4762 | | 6457 | |  |
| Rosuvastatin | C10AA07 | 6850 | 2482 | | 4368 | | 451 | | 6399 | | 2525 | | 4325 | | 322 | | 3225 | | 3464 | |  |
| Mometasone | D07AC13 | 7598 | 4270 | | 3328 | | 298 | | 7300 | | 2496 | | 5102 | | 4730 | | 3272 | | 1961 | |  |
| Erythromycin | D10AF02 | 866 | 624 | | 242 | | 14 | | 852 | | 285 | | 581 | | 1326 | | 167 | | 36 | |  |
| Dexamethasone | H02AB02 | 834 | 317 | | 517 | | 96 | | 738 | | 261 | | 573 | | 324 | | 382 | | 290 | |  |
| Nitrofurantoin | J01XE01 | 8477 | 4978 | | 3499 | | 391 | | 8086 | | 462 | | 8015 | | 6168 | | 3070 | | 2323 | |  |
| Encephalitis, tick borne, inactivated, whole virus | J07BA01 | 4368 | 2990 | | 1378 | | 108 | | 4260 | | 1716 | | 2652 | | 3430 | | 1882 | | 771 | |  |
| Alprazolam | N05BA12 | 8417 | 4662 | | 3755 | | 390 | | 8027 | | 2121 | | 6296 | | 4252 | | 4051 | | 2240 | |  |
| Zolpidem | N05CF02 | 3069 | 1483 | | 1586 | | 205 | | 2864 | | 890 | | 2179 | | 1224 | | 1451 | | 1006 | |  |
| Melatonin | N05CH01 | 187 | 98 | | 89 | | 8 | | 179 | | 59 | | 128 | | 134 | | 74 | | 46 | |  |
| Olopatadine | S01GX09 | 701 | 350 | | 351 | | 17 | | 684 | | 204 | | 497 | | 606 | | 257 | | 141 | |  |
| Inpatient visits |  | 24 483 | 12 088 | | 12 395 | | 2670 | | 21813 | | 8523 | | 15 960 | | 8418 | | 8669 | | 7396 | |  |
| Outpatient visits |  | 68 793 | 45 377 | | 23 416 | | 2666 | | 66 127 | | 28 214 | | 40 579 | | 25 989 | | 28 891 | | 13 913 | |  |

**Supplementary table 4.** Adjusted incident rate ratios (estimate (95% CI)) of incident and recurrent use of drugs in COVID-19 and RESP groups in Estonia 2020–2021. Statistically significant differences between two groups after Bonferroni correction are in bold.

| **Drug name; route of administration** | **ATC code** | **Incident use** | | | **Recurrent use** | | |
| --- | --- | --- | --- | --- | --- | --- | --- |
|  |  | **COVID-19** | **RESP** | **p-value** | **COVID-19** | **RESP** | **p-value** |
| metformin; oral | A10BA02 | **1.48 (1.30…1.69)** | **0.91 (0.77…1.06)** | **< 0.001** | 1.00 (0.96…1.04) | 1.03 (0.98…1.07) | 0.395 |
| rivaroxaban; oral | B01AF01 | **1.32 (1.17…1.49)** | **0.82 (0.71…0.94)** | **< 0.001** | 1.20 (1.13…1.27) | 1.08 (1.01…1.14) | 0.009 |
| trimetazidine; oral | C01EB15 | **2.26 (1.96…2.59)** | **0.98 (0.83…1.15)** | **< 0.001** | **1.36 (1.26…1.47)** | **0.99 (0.92…1.07)** | **< 0.001** |
| metoprolol; oral | C07AB02 | **1.50 (1.37…1.63)** | **0.96 (0.87…1.06)** | **< 0.001** | 1.08 (1.05…1.11) | 1.09 (1.06…1.12) | 0.537 |
| rosuvastatin; oral | C10AA07 | **1.29 (1.17…1.42)** | **0.92 (0.82…1.04)** | **< 0.001** | 1.04 (1.00…1.08) | 1.02 (0.98…1.06) | 0.536 |
| mometasone; topical | D07AC13 | **1.24 (1.13…1.36)** | **0.87 (0.79…0.97)** | **< 0.001** | 1.06 (1.00…1.12) | 0.98 (0.92…1.04) | 0.077 |
| erythromycin; topical | D10AF02 | **1.63 (1.28…2.05)** | **0.69 (0.50…0.93)** | **< 0.001** | **1.41 (1.15…1.71)** | **0.74 (0.57…0.94)** | **< 0.001** |
| dexamethasone; systemic | H02AB02 | 1.29 (1.05…1.59) | 0.93 (0.71…1.22) | 0.06 | **1.60 (1.38…1.85)** | **0.92 (0.75…1.12)** | **< 0.001** |
| nitrofurantoin; oral | J01XE01 | **1.22 (1.12…1.34)** | **0.88 (0.80…0.97)** | **< 0.001** | **1.20 (1.12…1.27)** | **0.98 (0.92…1.04)** | **< 0.001** |
| encephalitis, tick borne, inactivated, whole virus; systemic | J07BA01 | **2.00 (1.77…2.25)** | **1.34 (1.19…1.49)** | **< 0.001** | 1.84 (1.68…2.00) | 1.47 (1.35…1.60) | < 0.001 |
| alprazolam; oral | N05BA12 | **1.25 (1.11…1.39)** | **0.91 (0.82…1.01)** | **< 0.001** | 1.04 (0.99…1.08) | 1.08 (1.04…1.11) | 0.168 |
| zolpidem; oral | N05CF02 | **1.53 (1.31…1.77)** | **0.91 (0.77…1.08)** | **< 0.001** | 1.09 (1.03…1.15) | 1.03 (0.98…1.09) | 0.170 |
| melatonin; oral | N05CH01 | 3.02 (1.93…4.64) | 0.86 (0.42…1.62) | 0.002 | **4.06 (2.80…5.83)** | **0.56 (0.32…0.90)** | **< 0.001** |
| olopatadine; ophthalmic | S01GX09 | 2.00 (1.58…2.51) | 1.02 (0.74…1.39) | 0.007 | **1.93 (1.62…2.27)** | **0.90 (0.72…1.13)** | **< 0.001** |

**Supplementary table 5.** Adjusted incidence rate ratios (estimate (95% CI)) comparing incident and recurrent drug use in COVID-19 and RESP subgroup of Charlson Comorbidity Index in Estonia 2020–2021. Statistically significant differences between two groups after Bonferroni correction are in bold.

| **Drug name; route of administration** |  | **Charlson = 0** | | **p-value** | **Charlson > 0** | | **p-value** |
| --- | --- | --- | --- | --- | --- | --- | --- |
|  | **ATC code** | **COVID-19** | **RESP** |  | **COVID-19** | **RESP** |  |
| **Incident use** | | |  |  |  |  |  |
| metformin; oral | A10BA02 | 6.24 (4.88…7.95) | 3.18 (2.33…4.31) | < 0.001 | 0.90 (0.76…1.06) | 0.61 (0.50…0.74) | 0.003 |
| rivaroxaban; oral | B01AF01 | 1.45 (1.18…1.77) | 0.91 (0.72…1.15) | < 0.001 | 1.21 (1.03…1.40) | 0.78 (0.65…0.93) | < 0.001 |
| trimetazidine; oral | C01EB15 | **3.03 (2.45…3.72)** | **1.15 (0.88…1.49)** | **< 0.001** | **1.70 (1.41…2.04)** | **0.91 (0.73…1.11)** | **< 0.001** |
| metoprolol; oral | C07AB02 | **1.86 (1.63…2.11)** | **1.12 (0.98…1.29)** | **< 0.001** | **1.24 (1.10…1.39)** | **0.85 (0.74…0.96)** | **< 0.001** |
| rosuvastatin; oral | C10AA07 | 1.62 (1.40…1.86) | 1.21 (1.01…1.46) | 0.016 | 1.12 (0.99…1.26) | 0.76 (0.65…0.89) | < 0.001 |
| mometasone; topical | D07AC13 | 1.26 (1.12…1.42) | 0.91 (0.79…1.03) | < 0.001 | 1.18 (1.02…1.35) | 0.84 (0.72…0.98) | 0.002 |
| erythromycin; topical | D10AF02 | 1.55 (1.16…2.04) | 0.66 (0.44…0.94) | < 0.001 | 1.70 (1.11…2.50) | 0.77 (0.43…1.29) | 0.023 |
| nitrofurantoin; oral | J01XE01 | **1.32 (1.17…1.47)** | **0.91 (0.80…1.02)** | **< 0.001** | 1.07 (0.93…1.23) | 0.86 (0.74…0.99) | 0.027 |
| encephalitis, tick borne,  inactivated, whole virus; systemic | J07BA01 | 1.95 (1.68…2.25) | 1.39 (1.22…1.59) | <0.001 | **2.20 (1.80…2.67)** | **1.21 (0.98…1.49)** | **< 0.001** |
| alprazolam; oral | N05BA12 | 1.34 (1.16…1.54) | 0.92 (0.80…1.05) | < 0.001 | 1.10 (0.92…1.29) | 0.90 (0.77…1.05) | 0.096 |
| zolpidem; oral | N05CF02 | 1.50 (1.20…1.86) | 0.99 (0.79…1.23) | 0.010 | 1.49 (1.22…1.81) | 0.84 (0.66…1.07) | < 0.001 |
| **Recurrent use** | | |  |  |  |  |  |
| trimetazidine; oral | C01EB15 | **2.11 (1.83…2.43)** | **1.14 (0.97…1.32)** | **< 0.001** | 1.15 (1.05…1.26) | 0.95 (0.87…1.04) | 0.004 |
| erythromycin; topical | D10AF02 | 1.28 (1.00…1.61) | 0.76 (0.56…1.01) | 0.007 | 1.64 (1.17…2.24) | 0.67 (0.41…1.04) | 0.002 |
| dexamethasone; systemic | H02AB02 | 1.76 (1.36…2.25) | 1.45 (1.02…2.03) | 0.381 | **1.39 (1.16…1.65)** | **0.73 (0.56…0.93)** | **< 0.001** |
| nitrofurantoin; oral | J01XE01 | 1.20 (1.10…1.31) | 0.98 (0.90…1.07) | < 0001 | 1.17 (1.07…1.28) | 0.98 (0.89…1.07) | 0.005 |
| melatonin; oral | N05CH01 | 3.08 (1.73…5.29) | 0.79 (0.34…1.62) | 0.006 | **4.11 (2.55…6.51)** | **4.68 (2.68…8.07)** | **< 0.001** |
| olopatadine; ophthalmic | S01GX09 | 1.94 (1.49…2.49) | 0.91 (0.64…1.26) | < 0.001 | 1.87 (1.50…2.31) | 2.00 (1.51…2.62) | < 0.001 |

**Supplementary table 6.** Adjusted incidence rate ratios (estimate (95% CI)) comparing incident and recurrent drug use in COVID-19 and RESP subgroup of hospitalisation during acute infection phase in Estonia 2020–2021. Statistically significant differences between two groups after Bonferroni correction are in bold.

| **Drug name; route of administration** | **ATC code** | **Hospitalised during acute phase** | | |  | **Not hospitalised during acute phase** | | |  | |
| --- | --- | --- | --- | --- | --- | --- | --- | --- | --- | --- |
|  |  | **COVID-19** | **RESP** | | **p-value** | **COVID-19** | **RESP** | | **p-value** | |
| **Incident use** |  |  |  | |  |  |  | |  | |
| metformin; oral | A10BA02 | 2.23 (1.67…2.92) | 0.96 (0.49…1.71) | | 0.016 | 1.31 (1.13…1.52) | 0.90 (0.76…1.06) | | 0.001 | |
| rivaroxaban; oral | B01AF01 | 1.99 (1.57…2.50) | 1.25 (0.86…1.77) | | 0.033 | 1.10 (0.95…1.26) | 0.77 (0.66…0.90) | | 0.001 | |
| trimetazidine; oral | C01EB15 | 2.84 (2.03…3.89) | 1.01 (0.46…1.96) | | 0.010 | **2.09 (1.79…2.43)** | **0.98 (0.83…1.16)** | | **< 0.001** | |
| metoprolol; oral | C07AB02 | 2.51 (2.09…3.00) | 1.52 (1.15…1.98) | | 0.002 | **1.30 (1.18…1.43)** | **0.91 (0.82…1.01)** | | **< 0.001** | |
| rosuvastatin; oral | C10AA07 | 1.65 (1.31…2.05) | 1.26 (0.84…1.85) | | 0.254 | 1.23 (1.11…1.36) | 0.90 (0.79…1.01) | | < 0.001 | |
| mometasone; topical | D07AC13 | 1.72 (1.30…2.24) | 1.02 (0.59…1.65) | | 0.078 | **1.20 (1.08…1.32)** | **0.87 (0.78…0.96)** | | **< 0.001** | |
| erythromycin; topical | D10AF02 |  |  | |  | **1.61 (1.26…2.04)** | **0.65 (0.46…0.88)** | | **< 0.001** | |
| nitrofurantoin; oral | J01XE01 | 1.30 (0.98…1.70) | 1.00 (0.65…1.49) | | 0.310 | **1.21 (1.10…1.33)** | **0.88 (0.80…0.97)** | | **< 0.001** | |
| encephalitis, tick borne, inactivated, whole virus; systemic | J07BA01 | 2.03 (1.24…3.15) | 0.75 (0.29…1.61) | | 0.045 | **2.00 (1.77…2.26)** | **1.35 (1.20…1.51)** | | **< 0.001** | |
| alprazolam; oral | N05BA12 | 1.85 (1.34…2.50) | 1.03 (0.63…1.59) | | 0.038 | 1.18 (1.05…1.33) | 0.91 (0.81…1.01) | | 0.001 | |
| zolpidem; oral | N05CF02 | 3.14 (2.28…4.25) | 0.84 (0.41…1.57) | | <0.001 | 1.27 (1.07…1.51) | 0.92 (0.77…1.09) | | 0.008 | |
| **Recurrent use** |  |  |  | |  |  |  | |  | |
| trimetazidine; oral | C01EB15 | 1.51 (1.28…1.78) | 0.84 (0.63…1.09) | | < 0.001 | **1.31 (1.20…1.43)** | **1.01 (0.93…1.09)** | | **< 0.001** | |
| erythromycin; topical | D10AF02 |  |  | |  | **1.38 (1.13…1.68)** | **0.71 (0.54…0.90)** | | **< 0.001** | |
| dexamethasone; systemic | H02AB02 | **3.37 (2.59…4.41)** | **0.90 (0.54…1.43)** | | **< 0.001** | 1.13 (0.94…1.35) | 0.93 (0.74…1.16) | | 0.187 | |
| nitrofurantoin; oral | J01XE01 | 1.37 (1.15…1.62) | 1.42 (1.09…1.84) | | 0.913 | **1.17 (1.10…1.25)** | **0.96 (0.90…1.02)** | | **< 0.001** | |
| melatonin; oral | N05CH01 |  |  |  | | **3.44 (2.30…5.08)** | **0.56 (0.33…0.91)** | **< 0.001** | |  |
| olopatadine; ophthalmic | S01GX09 |  |  |  | | **1.94 (1.63…2.30)** | **0.90 (0.71…1.13)** | **< 0.001** | |  |

**Supplementary table 7.** Adjusted incidence rate ratios (estimate (95% CI)) comparing incident and recurrent drug use in COVID-19 and RESP subgroup of sex in Estonia 2020–2021. Statistically significant differences between two groups after Bonferroni correction are in bold.

| **Drug name; route of administration** |  | **Female** | |  | **Male** | | |  | |  |
| --- | --- | --- | --- | --- | --- | --- | --- | --- | --- | --- |
|  | **ATC code** | **COVID-19** | **RESP** | **p-value** | **COVID-19** | **RESP** |  | **p-value** | |  |
| **Incident use** | | | | | | | | |  |  |
| metformin; oral | A10BA02 | 1.54 (1.30…1.81) | 0.93 (0.76…1.14) | < 0.001 | 1.33 (1.07…1.63) | 0.86 (0.65…1.12) | | 0.014 | |  |
| rivaroxaban; oral | B01AF01 | **1.33 (1.13…1.56)** | **0.80 (0.66…0.96)** | **< 0.001** | 1.22 (1.01…1.46) | 0.87 (0.70…1.07) | | 0.019 | |  |
| trimetazidine; oral | C01EB15 | **2.29 (1.94…2.69)** | **0.95 (0.78…1.15)** | **< 0.001** | 1.89 (1.45…2.42) | 1.08 (0.80…1.44) | | 0.005 | |  |
| metoprolol; oral | C07AB02 | **1.46 (1.30…1.63)** | **0.85 (0.75…0.96)** | **< 0.001** | 1.48 (1.29…1.69) | 1.16 (1.00…1.35) | | 0.020 | |  |
| rosuvastatin; oral | C10AA07 | **1.42 (1.26…1.60)** | **0.88 (0.76…1.03)** | **< 0.001** | 1.13 (0.97…1.30) | 0.97 (0.80…1.16) | | 0.218 | |  |
| mometasone; topical | D07AC13 | **1.27 (1.14…1.42)** | **0.86 (0.76…0.97)** | **< 0.001** | 1.13 (0.97…1.31) | 0.92 (0.77…1.08) | | 0.071 | |  |
| erythromycin; topical | D10AF02 | 1.86 (1.39…2.46) | 0.86 (0.59…1.21) | < 0.001 | 1.23 (0.81…1.79) | 0.41 (0.20…0.74) | | 0.005 | |  |
| nitrofurantoin; oral | J01XE01 | **1.20 (1.09…1.32)** | **0.86 (0.78…0.94)** | **< 0.001** | 1.50 (1.06…2.06) | 1.48 (1.04…2.06) | | 0.960 | |  |
| encephalitis, tick borne, inactivated, whole virus; systemic | J07BA01 | **2.08 (1.78…2.42)** | **1.28 (1.10…1.48)** | **< 0.001** | 1.96 (1.63…2.35) | 1.42 (1.19…1.69) | | 0.013 | |  |
| alprazolam; oral | N05BA12 | 1.21 (1.06…1.37) | 0.92 (0.81…1.03) | 0.002 | 1.29 (1.04…1.58) | 0.89 (0.73…1.09) | | 0.012 | |  |
| zolpidem; oral | N05CF02 | 1.41 (1.17…1.69) | 0.82 (0.66…1.00) | < 0.001 | 1.67 (1.30…2.12) | 1.17 (0.88…1.53) | | 0.061 | |  |
| Recurrent use | | | | | | | | |  | |
| trimetazidine; oral | C01EB15 | 1.33 (1.21…1.45) | 1.04 (0.95…1.13) | < 0.001 | **1.41 (1.21…1.63)** | **0.86 (0.72…1.00)** | | **< 0.001** | |  |
| erythromycin; topical | D10AF02 | 1.48 (1.15…1.88) | 0.85 (0.62…1.13) | 0.004 | 1.26 (0.92…1.69) | 0.55 (0.34…0.85) | | 0.004 | |  |
| dexamethasone; systemic | H02AB02 | 1.39 (1.16…1.66) | 0.83 (0.65…1.06) | 0.001 | 1.78 (1.41…2.24) | 1.16 (0.81…1.65) | | 0.049 | |  |
| nitrofurantoin; oral | J01XE01 | **1.18 (1.10…1.26)** | **0.97 (0.90…1.03)** | **< 0.001** | 1.43 (1.11…1.81) | 1.26 (0.95…1.65) | | 0.510 | |  |
| melatonin; oral | N05CH01 | 2.96 (1.82…4.71) | 0.55 (0.25…1.06) | < 0.001 | **4.68 (2.68…8.07)** | **0.60 (0.27…1.15)** | | **< 0.001** | |  |
| olopatadine; ophthalmic | S01GX09 | **1.85 (1.50…2.27)** | **0.91 (0.69…1.17)** | **< 0.001** | 2.00 (1.51…2.62) | 0.88 (0.55…1.36) | | 0.002 | |  |

**Supplementary table 8.** Adjusted incidence rate ratios comparing incident and recurrent drug use in age groups in COVID-19 and RESP groups in Estonia 2020–2021. Statistically significant differences between two groups after Bonferroni correction are in bold.

| **Drug name; route of administration** |  | **Age: 18-39y** | |  | **Age: 40-64y** | |  | **Age: 65+ y** | |  |
| --- | --- | --- | --- | --- | --- | --- | --- | --- | --- | --- |
|  | **ATC code** | **COVID-19** | **RESP** | **p-value** | **COVID-19** | **RESP** | **p-value** | **COVID-19** | **RESP** | **p-value** |
| **Incident use** | | |  |  |  |  |  |  |  |  |
| metformin; oral | A10BA02 | 1.45 (1.01…2.02) | 1.09 (0.71…1.63) | 0.309 | 1.35 (1.12…1.63) | 0.74 (0.57…0.94) | < 0.001 | 1.66 (1.33…2.04) | 1.07 (0.82…1.36) | 0.009 |
| rivaroxaban; oral | B01AF01 | 1.42 (0.96…2.05) | 0.99 (0.58…1.59) | 0.255 | 1.22 (1.00…1.48) | 0.80 (0.63…1.01) | 0.008 | 1.36 (1.14…1.61) | 0.81 (0.66…0.98) | < 0.001 |
| trimetazidine; oral | C01EB15 | 2.85 (1.96…4.05) | 0.92 (0.51…1.58) | 0.001 | **2.37 (1.94…2.87)** | **0.99 (0.77…1.27)** | **< 0.001** | 1.84 (1.43…2.33) | 0.98 (0.77…1.23) | < 0.001 |
| metoprolol; oral | C07AB02 | 1.48 (1.16…1.87) | 1.03 (0.78…1.35) | 0.051 | 1.35 (1.19…1.54) | 0.98 (0.85…1.12) | 0.001 | **1.66 (1.44…1.90)** | **0.92 (0.79…1.07)** | **< 0.001** |
| rosuvastatin; oral | C10AA07 | 2.86 (1.82…4.37) | 1.82 (0.92…3.37) | 0.256 | 1.17 (1.03…1.32) | 0.84 (0.71…0.99) | 0.002 | 1.38 (1.18…1.61) | 0.96 (0.81…1.15) | 0.002 |
| mometasone; topical | D07AC13 | 1.14 (0.97…1.34) | 0.81 (0.67…0.97) | 0.005 | 1.30 (1.13…1.49) | 0.96 (0.82…1.11) | 0.004 | 1.27 (1.03…1.54) | 0.83 (0.67…1.01) | 0.004 |
| erythromycin; topical | D10AF02 | **1.74 (1.32….2.26)** | **0.69 (0.48…0.97)** | **< 0.001** | 1.31 (0.72…2.21) | 0.59 (0.25…1.22) | 0.111 | 1.24 (0.29…3.64) | 0.95 (0.22…2.92) | 0.774 |
| nitrofurantoin; oral | J01XE01 | 1.27 (1.10…1.45) | 0.97 (0.84…1.13) | 0.010 | **1.23 (1.06…1.43)** | **0.77 (0.66…0.90)** | **< 0.001** | 1.09 (0.89…1.33) | 0.91 (0.75…1.08) | 0.170 |
| encephalitis, tick borne, inactivated, whole virus; systemic | J07BA01 | 1.80 (1.48…2.17) | 1.23 (1.23…1.23) | < 0.001 | 2.19 (1.85…2.59) | 1.48 (1.25…1.74) | 0.001 | 2.02 (1.39…2.84) | 1.29 (1.28…1.29) | 0.013 |
| alprazolam; oral | N05BA12 | 1.28 (1.05…1.54) | 0.94 (0.78…1.13) | 0.026 | 1.15 (0.98…1.35) | 0.89 (0.76…1.03) | 0.023 | 1.41 (1.10…1.79) | 0.91 (0.72…1.13) | 0.008 |
| zolpidem; oral | N05CF02 | 1.48 (1.06…2.02) | 0.95 (0.66…1.31) | 0.061 | 1.37 (1.09…1.69) | 0.90 (0.70…1.14) | 0.012 | 1.83 (1.39…2.38) | 0.92 (0.68…1.23) | < 0.001 |
| **Recurrent use** | | |  |  |  |  |  |  |  |  |
| trimetazidine; oral | C01EB15 | 2.12 (1.52…2.91) | 0.71 (0.42…1.13) | < 0.001 | **1.78 (1.57…2.01)** | **1.02 (0.89…1.17)** | **< 0.001** | 1.10 (0.98…1.22) | 0.99 (0.90…1.09) | 0.166 |
| erythromycin; topical | D10AF02 | **1.46 (1.16…1.81)** | **0.69 (0.51…0.91)** | **< 0.001** | 1.30 (0.83…1.97) | 0.9 (0.50…1.51) | 0.299 | 1.11 (0.33…2.83) | 0.92 (0.21…2.79) | 0.829 |
| dexamethasone; systemic | H02AB02 | 1.74 (1.28…2.34) | 0.83 (0.50…1.32) | 0.011 | 1.51 (1.20…1.88) | 1.14 (0.87…1.49) | 0.125 | 1.63 (1.27…2.08) | 0.65 (0.43…0.95) | < 0.001 |
| nitrofurantoin; oral | J01XE01 | 1.26 (1.14…1.39) | 1.04 (0.93…1.15) | 0.008 | 1.20 (1.08…1.33) | 0.93 (0.84…1.04) | 0.001 | 1.10 (0.97…1.24) | 0.98 (0.87…1.09) | 0.182 |
| melatonin; oral | N05CH01 | 1.17 (0.39…2.83) | 0.37 (0.16…0.72) | 0.068 | **6.04 (3.61…10.07)** | **0.61 (0.20…1.47)** | **< 0.001** | 4.12 (2.02…8.11) | 1.50 (0.48…3.92) | 0.115 |
| olopatadine; ophthalmic | S01GX09 | 2.17 (1.71…2.75) | 1.01 (0.71…1.40) | < 0.001 | 1.81 (1.38…2.36) | 0.82 (0.54…1.2) | 0.001 | 1.39 (0.82…2.23) | 0.84 (0.50…1.35) | 0.165 |

**Supplementary table 9.** Adjusted incidence rate ratios comparing inpatient and outpatient visits of COVID-19 and RESP group in main and subgroups during post-COVID-19 period in Estonia 2020–2021. Statistically significant differences between two groups after Bonferroni correction are in bold.

|  | **Inpatient** | |  | **Outpatient** | |  |
| --- | --- | --- | --- | --- | --- | --- |
|  | **COVID-19** | **RESP** | **p-value** | **COVID-19** | **RESP** | **p-value** |
| All subjects | 1.17 (1.12…1.21)  N = 52 089 | 1.21 (1.17…1.25)  N = 49 534 | 0.162 | **1.14 (1.14…1.15)** | **1.12 (1.11…1.13)**  N = 2 480 195 | **< 0.001** |
| Charlson = 0 | 1.46 (1.38…1.54)  N = 18 818 | 1.49 (1.41…1.58)  N = 19 385 | 0.588 | **1.18 (1.17…1.19)** | **1.16 (1.15…1.17)** | **< 0.001** |
| Charlson > 0 | - 1. (0.96…1.05)   N = 33 271 | 1.04 (0.99…1.09)  N = 30 149 | 0.357 | **1.08 (1.07…1.08)** | **1.04 (1.03…1.05)** | **< 0.001** |
| Hospitalised in acute phase | 1.82 (1.70…1.94)  N = 15 090 | 1.10 (1.06…1.15)  N = 9257 | 0.870 | 1.12 (1.12…1.13) | 1.11 (1.10…1.11) | 0.945 |
| Non-hospitalised in acute phase | **1.00 (0.95…1.04)**  N = 36 999 | **1.83 (1.70…1.98)**  N = 40 277 | **< 0.001** | **1.28 (1.26…1.3)** | **1.28 (1.25…1.30)** | **< 0.001** |
| Male | **1.15 (1.08…1.21)**  N = 19 996 | **1.29 (1.22…1.36)**  N = 18 727 | **< 0.001** | 1.14 (1.13…1.15) | 1.15 (1.14…1.16) | 0.569 |
| Female | 1.15 (1.10…1.21)  N = 32 093 | 1.13 (1.08…1.18)  N = 30 807 | 0.500 | **1.12 (1.11…1.13)** | **1.08 (1.07…1.09)** | **< 0.001** |
| Age: 18-39 | 1.18 (1.10…1.26)  N = 14 441 | 1.27 (1.18…1.36)  N = 27 928 | 0.143 | **1.11 (1.10…1.12)** | **1.17 (1.16…1.18)** | **< 0.001** |
| Age: 40-64 | 1.11 (1.05…1.18)  N = 18 451 | 1.14 (1.08…1.22)  N = 16 799 | 0.517 | **1.14 (1.13…1.15)** | **1.09 (1.09…1.10)** | **< 0.001** |
| Age: 65+ | 1.18 (1.11…1.25)  N = 19 197 | 1.20 (1.13…1.27)  N = 18 771 | 0.733 | **1.17 (1.16…1.19)** | **1.09 (1.08…1.10)** | **< 0.001** |
